# Cost of Illness Due to Typhoid Fever in Pemba, Zanzibar, East Africa

**Published:** 2014-09

**Authors:** Arthorn Riewpaiboon, Moritz Piatti, Benedikt Ley, Jacqueline Deen, Kamala Thriemer, Lorenz von Seidlein, Mohammad Salehjiddawi, Clara Jana-Lui Busch, Wolfgang H. Schmied, Said Mohammed Ali, The Typhoid Economic Study Group (GiDeok Pak, Leon R. Ochiai, Mahesh K. Puri, Na Yoon Chang, Thomas F. Wierzba, and John D. Clemens)

**Affiliations:** ^1^Faculty of Pharmacy, Mahidol University, Bangkok, Thailand; ^2^Ministry of Health and Social Welfare, Zanzibar, Tanzania; ^3^International Vaccine Institute, Seoul, Korea; ^4^University of Vienna, Biocenter, Vienna, Austria; ^5^Menzies School of Health Research, Casuarina, NT, Australia; ^6^Public Health Laboratory (Pemba)–Ivo de Carneri, Chake-Chake, Tanzania; ^*^All were affiliated to the International Vaccine Institute, Seoul, Korea)

**Keywords:** Cost of illness, Incidence-based approach, Typhoid fever, Tanzania

## Abstract

The aim of this study was to estimate the economic burden of typhoid fever in Pemba, Zanzibar, East Africa. This study was an incidence-based cost-of-illness analysis from a societal perspective. It covered new episodes of blood culture-confirmed typhoid fever in patients presenting at the outpatient or inpatient departments of three district hospitals between May 2010 and December 2010. Cost of illness was the sum of direct costs and costs for productivity loss. Direct costs covered treatment, travel, and meals. Productivity costs were loss of income by patients and caregivers. The analysis included 17 episodes. The mean age of the patients, was 23 years (range=5-65, median=22). Thirty-five percent were inpatients, with a mean of 4.75 days of hospital stay (range=3-7, median=4.50). The mean cost for treatment alone during hospital care was US$ 21.97 at 2010 prices (US$ 1=1,430.50 Tanzanian Shilling─TSH). The average societal cost was US$ 154.47 per typhoid episode. The major expenditure was productivity cost due to lost wages of US$ 128.02 (83%). Our results contribute to the further economic evaluation of typhoid fever vaccination in Zanzibar and other sub-Saharan African countries.

## INTRODUCTION

As pneumococcal and *Haemophilus influenzae* type b vaccines are included into the Expanded Programme on Immunization (EPI) in many countries and the rates of *falciparum* malaria continue to decline, typhoid fever may emerge as one of the most important causes of severe febrile illness in sub-Saharan Africa. A recent comprehensive meta-analyses of bloodstream infections in Africa that included 22 studies and 58,296 hospitalized patients found that, of the 5,647 non-malaria pathogenic isolates, 560 (10%) were *S.* Typhi (1). It has been estimated that *Salmonella enterica* serotype Typhi (*S.* Typhi) causes about 33 to 233 new cases/100,000 population/year in Africa (2,3). In Pemba, Zanzibar, a prospective study calculated an adjusted rate for typhoid fever of 110 cases/100,000 population/year (4). There is evidence that, in urban areas of sub-Saharan Africa, rates of typhoid fever are even higher and are similar to those in Asian urban slums (5).

The lack of a specific clinical presentation of typhoid fever makes diagnosis difficult. Even where blood-culture facilities and appropriate antibiotics are available, case-fatality rates are high (6,7), underscoring the need for effective preventive strategies, such as vaccination. The emergence of multidrug-resistant *Salmonellae* further emphasizes the need for public health interventions. Licensed, safe, and effective vaccines to prevent typhoid fever are available. In order to determine the potential value of vaccination, information from a cost-effectiveness analysis (CEA) is needed. The majority of studies on the cost of typhoid fever (8-11) and CEA of typhoid vaccination (12-16) have been done in Asia. To our knowledge, no such economic study has been conducted in sub-Saharan Africa. We, therefore, conducted a cost analysis of typhoid fever in Pemba, Zanzibar, Tanzania.

## MATERIALS AND METHODS

### Study design

We performed an incidence-based cost-of-illness evaluation from a societal perspective, based on a bottom-up or microcosting approach (17). The incidence-based approach measures the economic burden on patients over the entire period of illnesses. It considers only new cases occurring within a study period and monitors them throughout the entire period of illnesses. Societal cost of illness is defined as a combination of direct medical costs, direct non-medical costs, and indirect costs. Direct medical costs are healthcare-related costs directly spent at the hospitals and other health facilities. Direct non-medical costs include travel, meal and accommodation costs. Indirect costs include the opportunity cost of time spent by patients and caregivers for informal care (17).

### Study site

Zanzibar is a semi-autonomous province of the United Republic of Tanzania. The Zanzibar archipelago consists of two main islands—Unguja (also known as Zanzibar) and Pemba—as well as several smaller islands. The islands are situated off the eastern coast of Tanzania mainland (Unguja about 40 km and Pemba 60 km) (18). The total area of Zanzibar is approximately 2,460 sq. km (Unguja consists of 1,658 sq. km and Pemba 984 sq. km). Unguja is generally flat and sandy whereas Pemba's terrain is hilly, fertile, and heavily vegetated. Pemba is more rural and less developed than Unguja. Pemba's main town is Chake-Chake and is located in the district of the same name. The population of Zanzibar was 981,754 in 2002, with an annual growth rate of 3.1% (18). Of over 1 million estimated people in 2006, 66% lived in Unguja and 34% in Pemba. The 2006 population of Chake-Chake district was estimated to be 91,288. The primary public healthcare sector in Zanzibar consists of 124 Public Healthcare Units that provide general healthcare at the peripheral level and 4 Public Heathcare Centres that provide a wider range of health services and can admit up to 30 inpatients. There are three district hospitals in Pemba and one referral hospital Mnazi Mmoja in Unguja. Healthcare is *de jure* free of charge for all. However, since healthcare facilities have been continuously underfunded, informal user fees are charged at most facilities to fill this gap. No public, private or community-based health insurance funds exist, and health providers are funded exclusively through the Ministry of Health. In 2010, public expenditure on health was US$ 18.8 million in the archipelago, distributed evenly across the two islands and subsidized by 60% from development partners. The current reform of the health sector includes the formalization of user fees and an inclusion of a health insurance package in the Zanzibar Social Security Fund (19).

This study was conducted in conjunction with a blood culture-based passive surveillance of community-acquired bloodstream infections, set up in all three district hospitals on Pemba island (Chake-Chake, Wete, and Mkoani) (4).

### Study sample

Patients with blood culture-confirmed typhoid fever were identified through surveillance (4) at the three district hospitals in Pemba. Inclusion criteria were: age above 2 months and a recorded temperature of ≥37.5 °C for outpatients or any history of fever for inpatients. Blood samples were taken for culture, malaria diagnosis, and additional bedside tests. Culture bottles were incubated in an automated blood culture machine (BACTEC 9050, BD, USA) and processed according to standard procedures. Colonies suspected to have for *S*. Typhi were confirmed by API20E (Biomerieux, France) and serology (Beckton & Dickinson, USA) as described in detail elsewhere (4). All blood culture-confirmed typhoid fever cases during the surveillance period from May 2010 to December 2010 were included in this study.

### Data collection and management

To collect data on medical service, outpatient and inpatient records were reviewed. To calculate costs outside the study hospitals, patients or family members in the case of minors were interviewed 5-7 days (depending how quick the laboratory diagnosis was available), 14, 30, and 90 days after enrollment in the study. The interview was conducted in local language (Kiswahili), using a structured questionnaire, and information on travel cost, meal cost, and time loss was collected. The questionnaire was designed in English, translated into Kiswahili, back-translated into English, and piloted on the site. Two local interviewers were trained on the concepts and methods of the study and on how to conduct the interview. All data were double-entered into a custom-made database, using FoxPro software (Microsoft, Seattle, Washington, USA) and then exported to Microsoft Excel (Microsoft, Seattle, Washington, USA) for verification and further analyses.

### Definitions and costing method

We defined total cost of illness as the sum of direct costs and costs for productivity loss. Direct costs were defined as the sum of expenditure for treatment, travel, and meals—before, during, and after hospital care. Productivity costs were calculated by multiplying the patient's and caregiver's absence from work, with the average income (mean per-capita household annual income) in Zanzibar, which was US$ 4.47 (6,400 TSH) per day in 2010 (20). For the sensitivity analysis, we used countrywide average income (US$ 1.26 or 1,811 TSH per day in 2010) in the calculation.

The costing method consisted of three steps: identifying, measuring, and valuing resources used (21). Resources used in this study included medical services from the study hospitals and other health facilities, travelling, and food consumed while receiving medical services and time loss of the patients and caregivers for informal care. Data on these resources were collected from patients’ medical records and through interviews with the patients or caregivers. Costs for travelling and meals were calculated from actual expenditure. Medical services were converted into monetary estimates by multiplying the amount of each resource used with the unit cost. The unit costs of outpatient and inpatient consultation and treatment for primary hospitals were derived from the WHO (US$ 1.27 per visit and US$ 5.06 per patient-day at 2010 prices) (22,23). The values were adjusted from 2005 to 2010 by consumer price index of medical care category. Drug prices were obtained from the Medical Stores Department of Tanzania (24). Unit costs of laboratory tests were the median prices from the study hospitals.

### Analysis

Descriptive statistics were used in summarizing the study variables. Differences in patient population per hospital were assessed applying chi-square and Fisher's exact test as appropriate, using Stata (version 10) (Stata Corporation, Texas, USA). A p value of <0.05 was considered significant. The cost results were presented in US dollar at 2010 value (US$ 1=1,430.50 TSH) (25). We conducted a one-way sensitivity analysis to assess the uncertainty of our findings. Country average income of US$ 1.26 or 1,811 TSH per day in 2010 (20) was included in the sensitivity analysis to explore the effect on the cost, comparing local-area income used as a base case.

### Ethics

This study was approved by the Institutional Review Board of the International Vaccine Institute and the Zanzibar Medical Research and Ethics Committee. All patients (or family members in the case of child patient) and caregivers participating in this study provided written informed consent.

## RESULTS

Seventeen laboratory-confirmed cases of typhoid fever were included in the study. We compared the patients’ characteristics, hospitalization, and recovery by district hospital (Table 1). Fifty-three percent of cases (9/17) were treated at Chake-Chake Hospital. Seventy-one percent were female. Six of the 17 (35%) patients were hospitalized (average 4.75 days) (Table 1 and 2). All 17 patients recovered. There were no significant differences observed across the patient groups per hospital for number of patients, age, gender, and admission (Table 1).

**Figure 1.**
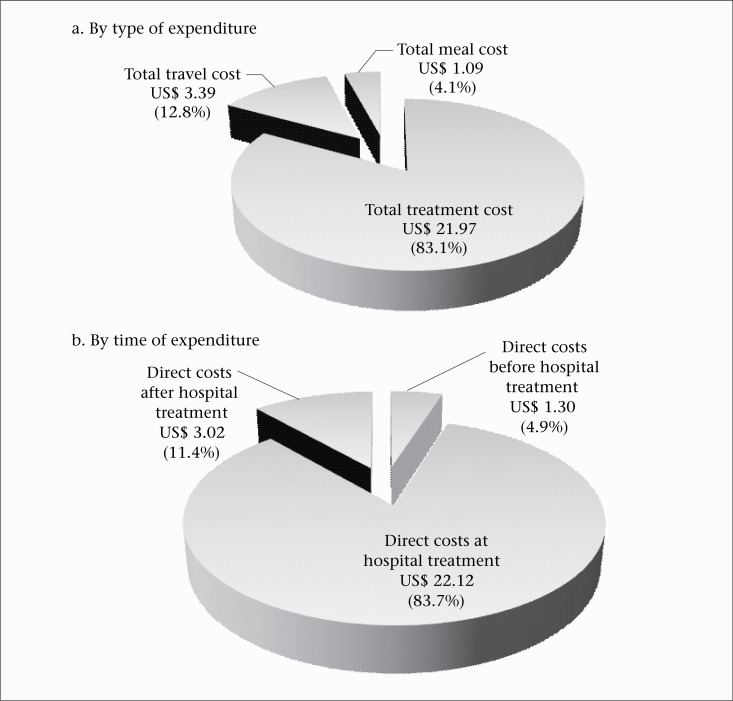
Breakdown of average direct cost

**Figure 2.**
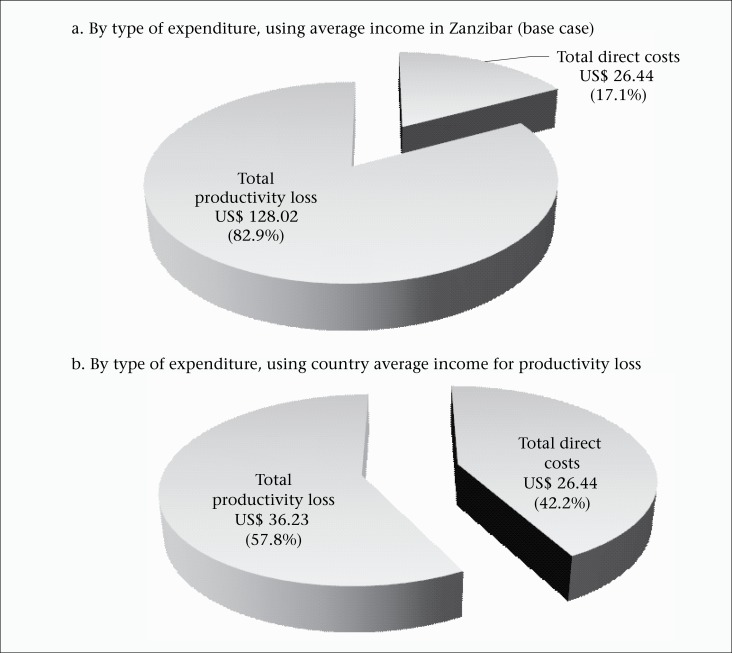
Breakdown of average cost of illness

Hospital services (cost per visit and patient-day), laboratory tests, and drugs used by the patients are presented in terms of unit costs (Table 3). The mean age of the patients included in the analysis was 23 years (Table 2). The overall mean cost of illness for a single episode of typhoid fever was US$ 154.47 (220,969 TSH) (Table 2). This comprised the direct costs before, during, and after hospitalization as well as productivity costs from lost wages. The direct cost per episode consisted of expenditure for treatment (US$ 21.97 or 31,429 TSH or 83%), travel (US$ 3.39 or 4,847 THS or 13%), and meals (US$ 1.09 or 1,553 TSH or 4%) before, during, and after hospitalization (Table 2). The largest proportion of expenditure was for the treatment costs during hospital care (Figure 1a and 1b). The mean productivity cost from lost wages was US$ 128.02 (183,139 TSH) (Table 2). This comprised 83% of the total cost of illness per episode [US$ 154.47 (220,969 TSH)] (Figure 2a). For the sensitivity analysis, we found that the proportions of expenditure changed. When the country average income (instead of Zanzibar average income) was used in the calculation of productivity cost, the productivity cost decreased to US$ 36.23. Accordingly, the average cost of illness was US$ 62.67 (median=US$ 52.86) (Figure 2b). Compared to the base case, productivity cost decreased from 83% to 58% per episode whereas direct costs increased from 17% to 42% (Figure 2b). For country extrapolation, adjusted incidence rates of *S*. Typhi were used (110 per 100,000 population) (4). Assuming an estimated population of Tanzania at 43,601,796 (26), the total number of typhoid cases would be 47,962 (43,601,796×110/100,000). We used the median of the cost of illness (US$ 52.86) calculated from the country average income to represent the country extrapolation. The overall economic burden caused by *S*. Typhi on the country was US$ 2,535,271 (US$ 52.86×47,962 cases). The 2010 per-capita GDP at current prices was US$ 551 (27), or 10 times the cost of falling ill. When aggregating across the nation, the total cost of *S.* Typhi amounted to 0.01% of GDP.

**Table 1.** Patients’ characteristics, type of treatment, and outcome

Characteristics	Chake-Chake	Mkoani	Wete	p	Total
Total number (%) of patients	9 (52.94)	5 (29.41)	3 (17.65)	0.085*	17 (100)
Mean (median) age in years	19.56 (22.00)	33.40 (23.00)	14.00 (11.00)	0.474***	23 (22)
No. (%) of females	7 (77.78)	4 (80.00)	1 (33.33)	0.384**	12 (70.59)
No. (%) of patients hospitalized	4 (44.44)	0 (0)	2 (66.67)	0.297**	6 (35.29)
No. (%) of patients recovered	9 (100)	5 (100)	3 (100)	NA	17 (100)

*Chi-square test;

**Fisher's exact test;

***Kruskal-Wallis test; NA=Not applicable

**Table 2.** Resource utilization and costs

Parameter	Mean	Median	Minimum	Maximum	SD
Age (years)	23	22	5	65	16
Length of hospital stay in days (among inpatients)	4.75	4.5	3	7	1.71
Costs before hospitalization in US$ (TSH)	1.29 (1,859)	0	0	6.99 (10,000)	2.13 (3,054)
Treatment	1.01 (1,44)	0	0	0.49 (7,000)	1.64 (2,344)
Travel	0.23 (329)	0	0	3.15 (4,500)	0.76 (1,094)
Meal	0.86 (88)	0	0	1.05 (1,500)	0.25 (364)
Costs during hospitalization in US$ (TSH)	22.12 (31,644)	15.63 (22,354)	8.64 (12,366)	77.91 (111,449)	17.06 (24,404)
Treatment	19.54 (27,956)	13.70 (19,596)	8.36 (11,966)	68.12 (97,449)	15.28 (21,865)
Travel	1.83 (2,624)	1.40 (2,000)	0	6.99 (10,000)	2.06 (2,944)
Meal	0.74 (1,065)	0.07 (100)	0	6.99 (10,000)	1.70 (2,439)
Costs after hospitalization in US$ (TSH)	3.02 (4,326)	0.98 (1,400)	0	14.44 (20,650)	4.56 (6,519)
Treatment	1.42 (2,032)	0	0	8.56 (12,250)	2.75 (3,936)
Travel	1.32 (1,894)	0.56 (800)	0	5.87 (8,400)	1.98 (2,826)
Meal	0.28 (400)	0	0	2.10 (3,000)	0.59 (837)
Total direct costs in US$ (TSH)	26.44 (37,829)	22.81 (32,629)	9.69 (13,866)	79.59 (113,849)	17.56 (25,123)
Treatment	21.97 (31,429)	18.90 (27,034)	9.15 (13,090)	68.12 (97,449)	14.88 (21,290)
Travel	3.39 (4,847)	1.96 (2,800)	0	10.14 (14,500)	3.63 (5,195)
Meal	1.09 (1,553)	0.35 (500)	0	7.90 (11,300)	1.96 (2,808)
No. of days of sickness before hospitalization	12	10	3	30	8
Total days of sickness	21	17	8	48	10
Children (up to 15 years)	26	24	14	48	12
Adult (>15 years)	17	16	8	30	8
Days spent in informal care	8	4	0	24	8
Total productivity loss in US$ (TSH)	128.02 (183,139)	120.79 (172,787)	44.74 (63,995)	319.86 (457,566)	68.01 (97,288)
Children (up to 15 years)	150.28 (215,752)	123.02 (175,980)	89.47 (127,987)	319.86 (457,560)	80.21 (114,743)
Adult (>15 years)	112.06 (160,309)	99.54 (142,392)	44.74 (64,001)	219.21 (313,580)	56.97 (81,502)
Total cost of illness in US$ (TSH)	154.47 (220,969)	138.28 (197,669)	54.43 (77,861)	399.45 (571,415)	82.55 (118,081)
Children (up to 15 years)	183.54 (262,556)	151.24 (216,349)	103.17 (147,585)	399.45 (571,413)	102.27 (146,298)
Adults (>15 years)	134.12 (191,586)	119.35 (170,723)	54.43 (77,862)	245.84 (351,674)	63.43 (90,732)

SD=Standard deviation

**Table 3.** Unit cost of medical services and selected drug items

Resource	Unit cost in US$ (TSH)
Outpatient routine service cost (per patient, per visit)	1.27 (1,817)
Inpatient hospital cost (per patient, per day)	5.12 (7,327)
Blood count	1.40 (2,000)
Blood chemistry	2.10 (3,000)
Ampicillin 500 mg vial injection	0.18 (254)
Ceftriaxone 250 mg vial injection	0.39 (557)
Ciprofloxacin 250 mg capsule	0.01 (19)
Ciprofloxacin 500 mg capsule	0.04 (53)
Glucose (5%) 500 mL	0.43 (618)
Paracetamol 120 mg/5 mL in 60-mL bottle	0.28 (404)
Paracetamol 500 mg tablet	0.01 (7)

## DISCUSSION

We calculated the cost of treatment for a typhoid fever episode in Pemba at US$ 21.97 (31,429 TSH). This is similar to results from a study in India that found the cost of treatment per episode of typhoid fever to be US$ 20.77 at 2004 prices (11). Although the costs of typhoid fever cannot be compared directly across sites due to differences in the time when the studies were conducted and variations in the prices of resources (e.g. salaries), the similarities supported our results.

Regarding the total cost of illness, we calculated US$ 154.47 (220,969 TSH) per episode in our study while US$ 71, US$ 126, and US$ 132 were calculated for the studies in Viet Nam, China, and Indonesia respectively (9). The main difference was in wage rate. Our study used average income in the calculations while the studies in those three countries used the actual income of respondents. Another difference lies in the methodology used. The costs from a societal perspective are composed of direct cost (treatment cost from hospital's perspective and direct non-medical cost from patient's perspective) and productivity loss (cost of time loss by patients and caregivers). In the abovementioned study (9), the total cost was composed of private and public costs. Private costs included out-of-pocket expenditure for treatment, meal, transportation and lodging expenses. Public costs consisted of treatment cost at the healthcare facilities. Public treatment cost was the result of a subtraction of treatment fee from costs incurred for providers. Therefore, treatment or direct medical costs cannot be compared.

Productivity cost accounted for 83% of the total cost. This, however, should be carefully interpreted. Productivity cost is the product of time absent from work due to illness and daily wage. In our study, the average duration of sickness was 21 days while the corresponding figures from the studies conducted in China, India, Indonesia, Pakistan, and Viet Nam ranged from 3 to 21 days (9). In this case analysis, the average income in Zanzibar was used while the study sites were all in Pemba, the northern island of the Zanzibar archipelago. Pemba has a considerable weaker economy than Unguja (the main island of Zanzibar). The average income in Zanzibar is driven up by income from the island of Unguja which has a more developed private sector and hosts the majority of civil servants. Hence, using the average income in Zanzibar may have introduced a bias towards higher cost of illness. In addition, for the sensitivity analysis, we applied the countrywide average income. However, income in Zanzibar was 3.5 times higher than the average country-level income (US$ 4.47 vs US$ 1.26 per person per day); the total cost of illness using income in Zanzibar, therefore, was 2.5 times higher than that from the country-level income (US$ 154.47 vs US$ 62.67).

The average monthly income in Zanzibar is approximately US$ 89.48 (TSH 128,000). A sudden shock expenditure of US$ 154.47 (TSH 220,969) for an episode of typhoid fever represents nearly worth two months of income. In the absence of a formal insurance scheme or social safety nets, the patient is left with two options: (i) to reduce current consumption or (ii) to borrow from friends and relatives and reduce future consumption. The consequences for the patient can be dire in terms of dynamic poverty entrapment. The theoretical possibility of such consequences has been explored extensively (28). However, the literature is yet to produce statistical evidence for such effects.

### Limitations

The key limitation of this study is the small sample-size of culture-confirmed typhoid fever cases. Seventeen patients allow for indicative figures only and make generalization across Zanzibar or sub-Saharan Africa difficult. The small sample-size impedes statistical significance, i.e. the probability that results occurred by random chance. Furthermore, the study sites were all in Pemba, which is quite different in terms of its geographic and demographic structure from the rest of Zanzibar. Therefore, countrywide extrapolation should be done with caution, especially taking into account the heterogenous nature of Tanzania in terms of varying incidence rates of typhoid fever across regions.

However, limitation relating to sample-size is common for studies on the cost of typhoid fever. Poulos *et al.* (9) based their calculation on 17, 58, 66, 79, and 107 cases in Viet Nam, China, Pakistan, India, and Indonesia respectively. Despite the described limitations, the findings in these studies were used for further analysis on cost-effectiveness of typhoid vaccination programme (29).

### Conclusions

This study is one of the few studies on the cost of typhoid globally and the first one in Africa. The results from this study could contribute to further economic evaluation of vaccination for typhoid fever in Zanzibar and other sub-Saharan African countries.

## ACKNOWLEDGEMENTS

We are grateful to the patients and their parents who made this work possible. We thank all technical staff and research assistants at the Public Health Laboratory, Pemba, particularly Amour Khamis Sakhiya and Mohammed Suleiman who conducted the interviews. This work was supported by a grant from the Swedish International Development Agency (Sida) through the International Vaccine Institute.
